# Insulin–Heart Axis: Bridging Physiology to Insulin Resistance

**DOI:** 10.3390/ijms25158369

**Published:** 2024-07-31

**Authors:** Alfredo Caturano, Raffaele Galiero, Erica Vetrano, Celestino Sardu, Luca Rinaldi, Vincenzo Russo, Marcellino Monda, Raffaele Marfella, Ferdinando Carlo Sasso

**Affiliations:** 1 Department of Advanced Medical and Surgical Sciences, University of Campania Luigi Vanvitelli, 80138 Naples, Italy; alfredo.caturano@unicampania.it (A.C.); raffaele.galiero@unicampania.it (R.G.); erica.vetrano@unicampania.it (E.V.); celestino.sardu@unicampania.it (C.S.); raffaele.marfella@unicampania.it (R.M.); 2 Department of Experimental Medicine, University of Campania Luigi Vanvitelli, 80138 Naples, Italy; marcellino.monda@unicampania.it; 3 Department of Medicine and Health Sciences “Vincenzo Tiberio”, Università degli Studi del Molise, 86100 Campobasso, Italy; luca.rinaldi@unimol.it; 4 Department of Biology, College of Science and Technology, Sbarro Institute for Cancer Research and Molecular Medicine, Temple University, Philadelphia, PA 19122, USA; v.p.russo@libero.it; 5 Division of Cardiology, Department of Medical Translational Sciences, University of Campania Luigi Vanvitelli, 80138 Naples, Italy

**Keywords:** insulin signaling, insulin resistance, insulin–cardiac axis, diabetic cardiomyopathy, cardiac hypertrophy

## Abstract

Insulin signaling is vital for regulating cellular metabolism, growth, and survival pathways, particularly in tissues such as adipose, skeletal muscle, liver, and brain. Its role in the heart, however, is less well-explored. The heart, requiring significant ATP to fuel its contractile machinery, relies on insulin signaling to manage myocardial substrate supply and directly affect cardiac muscle metabolism. This review investigates the insulin–heart axis, focusing on insulin’s multifaceted influence on cardiac function, from metabolic regulation to the development of physiological cardiac hypertrophy. A central theme of this review is the pathophysiology of insulin resistance and its profound implications for cardiac health. We discuss the intricate molecular mechanisms by which insulin signaling modulates glucose and fatty acid metabolism in cardiomyocytes, emphasizing its pivotal role in maintaining cardiac energy homeostasis. Insulin resistance disrupts these processes, leading to significant cardiac metabolic disturbances, autonomic dysfunction, subcellular signaling abnormalities, and activation of the renin–angiotensin–aldosterone system. These factors collectively contribute to the progression of diabetic cardiomyopathy and other cardiovascular diseases. Insulin resistance is linked to hypertrophy, fibrosis, diastolic dysfunction, and systolic heart failure, exacerbating the risk of coronary artery disease and heart failure. Understanding the insulin–heart axis is crucial for developing therapeutic strategies to mitigate the cardiovascular complications associated with insulin resistance and diabetes.

## 1. Introduction

Insulin signaling is crucial for regulating cellular metabolism, growth, and survival pathways. Insulin receptors are present in various tissues throughout the body. While most research on insulin signaling has focused on tissues involved in systemic metabolic regulation, such as adipose tissue, skeletal muscle, liver, and brain, it also plays a significant role in other organs, including the heart [1].

The heart, as a muscular pump, requires a vast amount of ATP to power its contractile machinery and ionic pumps. Consequently, myocardial metabolism is largely influenced by the availability of substrates. Changes in circulating insulin levels due to daily rhythms and feeding patterns directly affect cardiac metabolism. This regulation occurs both through insulin’s modulation of peripheral tissues, which influence myocardial substrate supply, and through its direct effects on the heart muscle itself [1,2].

Although insulin signaling in cardiomyocytes is not essential for maintaining cardiac metabolism under non-stressed conditions, various studies have shown that it has roles beyond metabolic regulation [1,2]. This review aims to explore the insulin–heart axis, highlighting the diverse functions of insulin in the heart, and its impact on health and disease.

## 2. Insulin Signaling and Metabolic Regulation in Cardiac Function

The human heart demands a significant amount of energy, relying on a steady flow of nutrients and oxygen to keep intracellular ATP levels stable, which is crucial for the constant cycles of heart muscle contraction and relaxation. With mitochondria occupying one-third of the cell volume in cardiac myocytes—the highest mitochondrial content of any cell type—the heart boasts a robust metabolic system. This is evidenced by the heart’s exceptional oxygen consumption rate per unit weight. Over the course of a day, the heart generates and utilizes approximately 3.5 to 5 kg of ATP, an amount 15 to 20 times its own weight [1,3,4].

The heart’s metabolic versatility allows it to utilize all classes of energy substrates—including carbohydrates, lipids, amino acids, and ketone bodies—for ATP production in the mitochondria. The primary source of this ATP is the mitochondrial oxidation of long-chain fatty acids (LCFAs), which provide about 60–70% of the heart’s energy needs. Additionally, glucose contributes around 20%, and lactate accounts for roughly 10% of the energy production under normal physiological conditions. In a healthy heart, mitochondria are mainly fueled by fatty acyl-coenzyme A (CoA) and pyruvate, the primary metabolites of fatty acids and carbohydrates, respectively. This metabolic flexibility is crucial for meeting the heart’s energy demands, especially during increased workloads or stress [1,5,6,7]. The cardiac myocytes’ reliance on oxidative phosphorylation within the mitochondria underscores the importance of a constant oxygen supply, which is facilitated by a dense network of coronary arteries and capillaries [1,8]. Additionally, the heart’s ability to rapidly adjust to increased energy demands is supported by the dynamic regulation of key enzymes involved in metabolic pathways, such as AMP-activated protein kinase (AMPK) and peroxisome proliferator-activated receptor alpha (PPARα), which play critical roles in maintaining energy homeostasis [9,10]. Furthermore, during prolonged fasting, the metabolic environment shifts significantly, leading to increased reliance on ketone bodies as an energy source for the heart. As glucose availability diminishes, the liver enhances the production of ketone bodies, particularly β-hydroxybutyrate and acetoacetate, from the oxidation of fatty acids. These ketone bodies become a crucial fuel for the heart due to their efficiency and abundance. The heart adapts to this metabolic shift by upregulating enzymes involved in ketone body utilization, such as 3-oxoacid CoA-transferase and β-hydroxybutyrate dehydrogenase. This adaptation allows the heart to maintain ATP production and function despite the low availability of glucose. Ketone bodies are more oxygen-efficient compared to fatty acids, yielding more ATP per molecule of oxygen consumed, which is advantageous during periods of limited nutrient intake. Thus, ketone bodies support cardiac energy metabolism effectively during prolonged fasting when fatty acid oxidation predominates and glucose is scarce [11].

**Figure 1:**
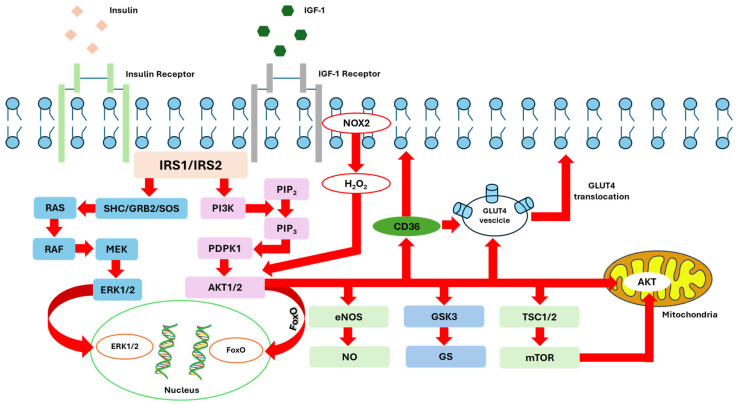
Schematic representation of the signaling intermediates involved in the pathways activated by the insulin receptor or insulin-like growth factor 1 (IGF-1) receptor in cardiomyocytes. This figure illustrates the cascade of molecular events initiated by the binding of insulin or IGF-1 to their respective receptors on the cardiomyocyte membrane. Upon ligand binding, the IR and IGF-1R undergo autophosphorylation, creating docking sites for insulin receptor substrates (IRS). The phosphorylated IRS proteins activate the ERK1/2, which translocate into the nucleus where they regulate gene expression by phosphorylating transcription factors involved in cell proliferation and differentiation, as well as activating the PI3K (phosphoinositide 3-kinase) pathway, leading to the generation of PIP3 (phosphatidylinositol-3,4,5-trisphosphate) and subsequent activation of Akt (protein kinase B). Activated Akt phosphorylates multiple downstream targets involved in glucose metabolism, protein synthesis, and cell survival. This includes the phosphorylation and inhibition of GSK-3β (glycogen synthase kinase-3 beta), promoting glycogen synthesis, the activation of mTOR (mechanistic target of rapamycin), which facilitates protein synthesis and cell growth; the activation of endothelial nitric oxide synthase (eNOS), leading to increased nitric oxide production, which is crucial for vascular function; activation of transcription factors such as FoxO, promoting their translocation into the nucleus, where they regulate gene expression involved in cell survival and metabolism; Akt mitochondrial translocation, which enhances mitochondrial function; and biogenesis, contributing to improved cellular energy homeostasis.

Cardiomyocytes have high levels of insulin receptors (IRs) and similarly express the insulin-like growth factor 1 receptor (IGF1R). These receptors activate overlapping signaling pathways within the heart (Figure 1) [12]. Both the IR and IGF1R connect with insulin receptor substrates (IRS1 and IRS2), which act as central nodes to relay insulin signals to downstream pathways. This signaling primarily involves phosphoinositide-3-kinase (PI3K) and protein kinase B (PKB/Akt), along with components of the extracellular signal-regulated kinase (ERK) pathway. When activated, Akt phosphorylates several downstream targets, such as tuberous sclerosis complex 2 (TSC2) and proline-rich Akt substrate 40 kDa (Pras40), thereby promoting the activity of the mechanistic target of rapamycin (mTOR) [1,13]. Additionally, Akt phosphorylates glycogen synthase kinase-3 (GSK3), thereby regulating glycogen metabolism and promoting cell survival pathway [14]. The PI3K/Akt pathway includes several classes of PI3Ks, each with distinct structures and activation modes. Class I PI3Ks are further divided into IA and IB categories based on their binding subunits. Class IA PI3K consists of catalytic subunits (p110α, p110β, p110δ) and a regulatory subunit (p85α), while class IB PI3K comprises the catalytic subunit p110γ, regulated by the protein p101. Class IB PI3K can be activated by G-protein-coupled receptors (GPCRs) via the β and γ subunits [15]. This activation cascade leads to the synthesis of phosphatidylinositol-3,4,5-trisphosphate (PIP3), which recruits phosphoinositide-dependent kinase-1 (PDK1) and Akt, culminating in Akt phosphorylation and activation. The full activation of Akt requires phosphorylation at Thr^308^ and Ser^473^, and Akt itself may autophosphorylate or be activated independently of PI3K [15]. Moreover, Akt phosphorylation leads to the nitric oxide synthase isoform 3 (NOS3) activation, which in turn generates nitric oxide (NO), which along with cyclic guanosine monophosphate (cGMP) activation plays a crucial role in cardiac physiology. Anti-apoptotic mediators such as the BCL2-associated agonist of cell death (BAD) and members of the forkhead box O (FOXO) family of transcriptional regulators are also integral to these pathways. These molecules work in concert to modulate diverse cellular processes in cardiomyocytes, including metabolism, cell growth, survival, and the suppression of apoptosis and autophagy [15,16]. Additionally, novel mechanisms include the requirement for NOX2/4-generated reactive oxygen species (ROS) and the regulation of ion channel function, highlighting the complexity and adaptability of these signaling networks [17]. For instance, class I PI3Ks are pivotal in these processes, and mammalian genomes encode three Akt isoforms—Akt1, Akt2, and Akt3—each with distinct roles in cellular function. Akt is central to many cardiovascular functions, including cell proliferation and growth via mTORC1, cell survival via caspase-9, YAP, Bcl-2, and Bcl-x activities, and angiogenesis and vasorelaxation via VEGF secretion and eNOS phosphorylation [18].

The heart’s substrate preference is influenced by the cardiac environment, including coronary flow, blood substrate supply, hormones, and nutritional status [19,20]. The Randle cycle explains how LCFA oxidation inhibits glucose uptake and catabolism [5]. During starvation or conditions such as chronic heart failure and poorly controlled diabetes, ketone bodies rise and become major substrates for the heart, inhibiting the oxidation of other substrates [21,22]. Conversely, when glucose and insulin concentrations rise, glucose becomes the preferred substrate. Insulin signaling, which includes the translocation of glucose transporter GLUT4 to the sarcolemmal membrane, enhances glucose uptake in cardiomyocytes [7].

Activation of the IR involves the binding of insulin to its extracellular α-subunits, leading to the activation of the intrinsic tyrosine kinase activity of the β-subunits and subsequent auto transphosphorylation. The IR shares a similar structure with the IGF-1 receptor, facilitating cross-reaction and overlapping functions [23,24]. Once phosphorylated, IR binds and phosphorylates downstream elements, including the insulin receptors family and SHC, leading to the activation of the PI3K and MAPK pathways [25,26,27,28]. PI3K, especially class Ia, is a crucial player in the metabolic actions of insulin, while the MAPK pathway is involved in cell growth and differentiation. The PI3K pathway culminates in the activation of PKB/Akt, with PDK1 being essential for this process in the heart. PKB/Akt is pivotal in regulating glucose uptake and other metabolic processes, including the translocation of GLUT4 [29,30,31,32]. However, Akt isoforms exhibit differential roles in myocardial metabolism. For instance, while Akt2 deletion reduces insulin-stimulated glucose uptake and ischemia tolerance, Akt1 phosphorylation remains unaffected under certain conditions [33,34]. Interestingly, in vitro studies using siRNA-mediated gene silencing demonstrate a specific role for Akt2 in cardiomyocyte glucose uptake. In diabetic models, impaired myocardial glucose utilization correlates with reduced Akt2 phosphorylation, contrasting with unchanged Akt1 phosphorylation [34].

It is noteworthy that GLUT4 translocation is induced by cardiac muscle contraction, leading to a less pronounced increase in glucose uptake during insulin-stimulated contraction compared to cultured cardiomyocytes or skeletal muscle and adipose tissue [2]. Additionally, genetic deletion of insulin receptors in the heart increases GLUT4 content and basal glycolysis rates, similar to insulin-perfused wild-type hearts, suggesting a minor direct role of insulin in regulating myocardial glucose uptake in vivo [2]. However, GLUT4-mediated glucose uptake in the heart plays a critical role in responding to ischemia and acute hemodynamic stress, as evidenced by studies on mice lacking cardiomyocyte GLUT4 [35,36,37].

Moreover, acute insulin stimulation provides cardioprotection during reperfusion following myocardial ischemia through pathways involving PI3K, Akt, and PKC activation [38,39,40]. Interestingly, the interplay between insulin signaling and ischemic preconditioning (IPC) is complex, as insulin’s protective effects against ischemia/reperfusion (I/R) injury can negate the benefits of IPC, which relies on Akt activation—a phenomenon observed with transgenic Akt overexpression in cardiomyocytes [41].

Insulin also induces the translocation of the LCFA transporter FAT/CD36 to the plasma membrane, promoting LCFA uptake [42,43,44,45]. Despite increased intracellular LCFA concentrations, insulin does not necessarily enhance LCFA oxidation but promotes storage in the intracellular lipid pool. Alongside GLUT4, other proteins like PIKfive, synip, AS160, and TBC1D1 are pivotal in insulin-mediated glucose uptake [46,47]. Furthermore, insulin stimulates glycogen synthesis and glycolysis by activating glycogen synthase and PFK-2, respectively, which amplifies glycolytic flux via fructose 2,6-bisphosphate production [48,49,50,51].

In vivo, the heart predominantly utilizes fatty acids (FAs) as its primary metabolic substrate during fasting, reflecting heightened FA availability due to increased lipolysis under low insulin conditions [52]. Conversely, under conditions of euglycemic hyperinsulinemia, myocardial glucose uptake surpasses that of FAs, which are concurrently suppressed in circulation [53,54]. Interestingly, despite increased insulin levels post-feeding, the heart utilizes absorbed FAs, underscoring the dynamic substrate preference dictated by physiological state [55,56]. Classic studies underscore insulin’s direct regulation of myocardial glucose metabolism [57,58].

Insulin enhances GLUT4 translocation in isolated hearts, activating PFK2 and augmenting glucose oxidation more than glycolysis alone, owing to pre-existing elevated levels of myocardial glycolysis independent of insulin [59,60]. Insulin also boosts glucose oxidation by mitochondrial Akt targeting, concomitantly suppressing mitochondrial FA oxidation via Randle’s cycle reversal. Additionally, insulin likely inhibits FAO by suppressing AMPK activity, thereby decreasing ACC phosphorylation and elevating malonyl CoA levels [61,62]. Despite these suppressive effects on FAO, insulin signaling promotes FA uptake by facilitating CD36 translocation to the sarcolemma through Akt2 and PKC-zeta isoform pathways, concurrently regulating GLUT4 trafficking [63,64]. Ultimately, short-term insulin stimulation directs FAs toward synthetic pathways like triglyceride production, crucial for the heart’s energetic demands [65].

## 3. Insulin Signaling and the Regulation of Physiological Cardiac Hypertrophy

Insulin signaling plays a crucial role in the regulation of physiological cardiac hypertrophy through a series of intricate molecular mechanisms. As an anabolic hormone, insulin promotes protein synthesis and cell growth primarily through the phosphorylation and dephosphorylation of various translation factors and ribosomal proteins, with PKB/Akt being a key regulator [66,67]. PKB/Akt phosphorylates and inactivates the TSC2, leading to the activation of the G protein Rheb and subsequently the mammalian target of rapamycin (mTOR) [68,69,70,71]. Activated mTOR then regulates protein translation by targeting 4E-BP1 and p70S6K, enhancing translational capacity and ribosomal biogenesis [72,73]. Additionally, PKB/Akt modulates glycogen synthase kinase-3 (GSK-3) and the forkhead transcription factor FOXO family, which are involved in protein synthesis and atrophy prevention, respectively [67]. The inhibition of GSK-3 by insulin stimulates the initiation of protein synthesis, and the phosphorylation of FOXOs by PKB/Akt prevents muscle atrophy by promoting their nuclear exclusion [29,74].

Furthermore, studies have shown that the absence of insulin signaling during heart development reduces heart size, highlighting its role in cardiac growth [32,75]. Exercise-induced physiological hypertrophy is mediated by PI3K and Akt signaling, which coordinate hypertrophic responses and mitochondrial adaptations characterized by increased oxidative capacity. However, persistent Akt activation can lead to pathological hypertrophy, indicating a delicate balance in insulin signaling pathways [76]. The cross-talk between insulin and IGF1 signaling also contributes to physiological cardiac hypertrophy, with defects in IGF1 signaling exacerbating exercise-induced hypertrophy, as evidenced by studies on mice with cardiomyocyte-specific deletions of IGF1R and IRS. IRS1 and IRS2 play nonredundant roles in the hypertrophic and bioenergetic responses to exercise, emphasizing the multifaceted nature of insulin signaling in cardiac physiology [76].

## 4. Pathophysiology of Insulin Resistance

Insulin resistance (IR) is a pathophysiological condition characterized by the diminished ability of insulin to exert its normal biological effects, particularly facilitating glucose entry into insulin-sensitive tissues to be used as the primary energy substrate. Diverse defects in signal transduction contribute to IR [77,78]. 

### 4.1. Pathophysiological Mechanisms of Proximal Insulin Signaling Impairment

**Figure 2:**
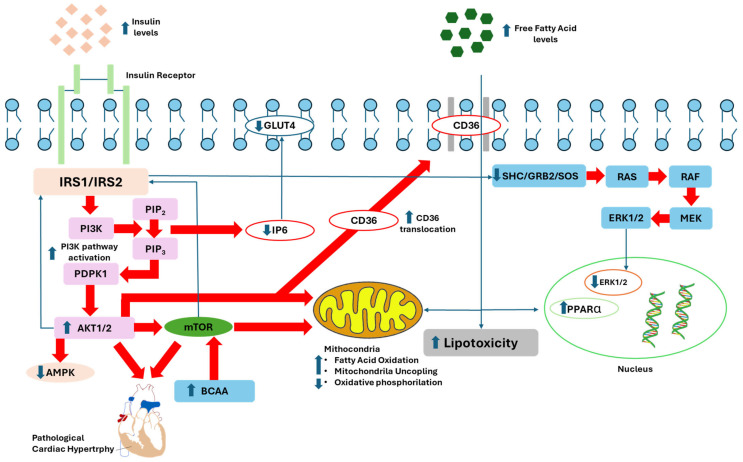
Schematic representation of the signaling intermediates involved in the pathways activated in an insulin-resistant state. Hyperinsulinemia triggers the activation of the phosphoinositide 3-kinase (PI3K), protein kinase B (Akt), and mechanistic target of rapamycin (mTOR) pathways within cardiac tissue. This activation drives cardiac hypertrophy and remodeling. The stimulation of this signaling cascade leads to the inhibition of insulin receptor substrate 1 (IRS1). Concurrently, the activation of Akt facilitates the translocation of CD36, enhancing fatty acid uptake by the heart. This process contributes to lipotoxicity and elevates mitochondrial fatty acid utilization. Additionally, it induces transcriptional modifications in the nucleus, resulting in altered gene expression within myocardial cells. Moreover, in insulin-resistant states, increased serine phosphorylation of IRS1 and other inhibitory modifications impair the ability of SHC and GRB2 to facilitate Ras-MAPK pathway activation. Furthermore, despite the increased activation of Akt, the translocation of glucose transporter member 4 (GLUT4) is hindered, likely due to a decrease in inositol hexakisphosphate (IP6).

Proximal insulin signaling impairment is a critical factor contributing to metabolic and cardiovascular disorders. This impairment often originates from disruptions in the early steps of the insulin signaling pathway, including the interaction of insulin with its receptor and the subsequent activation of downstream signaling molecules (Figure 2). 

The proximal insulin signaling pathway includes two primary branches: the metabolic branch, which is triggered by IRS proteins and SH2B2/APS and the mitogenic branch, which is initiated by GRB2 and SHC [79]. 

Unlike many other receptor tyrosine kinases that directly phosphorylate cytoplasmic substrates, the INSR recruits a variety of phosphotyrosine-binding proteins, allowing early diversification of insulin signaling to activate multiple functional modules [80]. For instance, SHC interacts through its phosphotyrosine-binding domain with INSR at pTyr^972^, while SH2B1, SH2B2/APS, GRB10, and GRB14 engage through their Src homology 2 (SH2) domains with the activated INSR activation loop. These interactions are crucial for regulating insulin signaling. For example, GRB10 phosphorylation and stabilization by mTORC1, activated by insulin signaling, provides feedback inhibition of INSR activity [81].

In metabolic disorders such as obesity and diabetes, individuals often exhibit reduced surface insulin receptor (INSR) content and diminished INSR kinase (IRK) activity, which are essential for proper insulin signaling [82]. Defective IRK activity is closely linked to decreased tyrosine phosphorylation of IRS1, a condition frequently observed in insulin-resistant skeletal muscles [83]. Targeted knockout or ablation of INSR in the liver results in the inability of insulin to suppress hepatic glucose production (HGP), highlighting the critical role of INSR in hepatic insulin resistance [84,85]. Additionally, reduced expression or increased serine phosphorylation of IRS proteins can impair their interaction with PI3K, subsequently downregulating PI3K activation and contributing to insulin resistance [86,87]. Mice models with homozygous deletions of the IRS1 or IRS2 genes display peripheral insulin resistance and diabetes, along with compromised insulin secretion due to disrupted PI3K/AKT signaling [88]. Upon insulin binding to INSR, autophosphorylation of the receptor occurs, which subsequently phosphorylates IRS proteins on tyrosine residues. These phosphorylated IRS proteins serve as docking sites for the p85 regulatory subunit of PI3K, leading to the activation of the p110 catalytic subunit of PI3K. This activation converts PIP2 to PIP3, which in turn recruits and activates PDK1 and AKT. Activated AKT then phosphorylates a variety of downstream targets involved in glucose uptake, glycogen synthesis, and lipid metabolism [89,90]. Consequently, pharmacological inhibitors, blocking antibodies, and PI3K knockdown abolish insulin stimulation of glucose transport, GLUT4 translocation, and DNA synthesis [91,92,93]. Deletion of Pik3r1 and Pik3r2, which encode PI3K subunit isoforms in skeletal muscle, impairs insulin-stimulated glucose transport [94]. Interference with Akt mutants similarly suppresses insulin-stimulated GLUT4 translocation, and reduced AKT expression or impaired AKT Ser473 phosphorylation is evident in insulin-resistant muscle and liver tissues [95,96,97,98,99]. Of the three known Akt isoforms (Akt1, Akt2, and Akt3) in insulin-sensitive tissues, defects in Akt2 and Akt3 particularly impair insulin-stimulated glucose transport in insulin resistance [100]. Elevated levels of plasma non-esterified fatty acids (NEFAs) can further impede the insulin-induced increase in IRS-1-associated PI3K activity without affecting Akt phosphorylation [101].

### 4.2. Pathophysiological Mechanisms of Distal Insulin Signaling Impairment

Evidence suggests that the PI3K-Akt/PKB pathway is one of the primary distal critical effectors in insulin signaling [102]. Akt/PKB activation induces a variety of downstream responses, including the translocation of glucose transporters (GLUTs) to the cell membrane, thereby increasing glucose uptake. More than one hundred Akt substrates have been identified, such as GLUT4, FOXO1, GSK3, mTORC1, SREBP-1c, ABHD15, TSC1/2, PDE3B, and PRAS40 [77,103]. Among these, GLUT4 is essential for glucose uptake in skeletal muscle and adipose tissue following insulin stimulation [104,105]. Impaired translocation of GLUT4 storage vesicles results in decreased insulin-stimulated glucose uptake, contributing to insulin resistance in both muscle and adipose tissues [106]. This impairment is seen in various models of insulin resistance and in humans with type 2 diabetes mellitus (T2DM). Heterozygous deletion of GLUT4 in mice reduces glucose uptake and leads to metabolic dysfunction in adipocytes, while defective insulin-stimulated GLUT4 translocation is seen in skeletal muscle in various IR mouse models and humans with T2DM [107,108,109,110,111,112]. The loss of Tbc1d4, an Akt substrate that regulates Rab-GTPase proteins associated with GLUT4 vesicles, significantly impairs insulin-stimulated glucose uptake in adipocytes [113]. Mice with a knockin mutation in TBC1D4 exhibit defective insulin-stimulated GLUT4 translocation in myocytes, resulting in glucose intolerance [114]. Inositol pyrophosphates, synthesized by inositol hexakisphosphate (IP6) kinase 1 (IP6K1), compete with PIP3 for binding to the pleckstrin homology (PH) domain of Akt/PKB, thereby inhibiting Akt signaling [115]. IP6K1 knockout mice show increased insulin sensitivity and resistance to obesity, indicating a novel therapeutic target for combating insulin resistance [116]. Recent studies have also indicated that chronic inflammation and endoplasmic reticulum stress contribute to the dysregulation of insulin signaling pathways, exacerbating insulin resistance [117,118,119]. Moreover, IP6 itself has been shown to exert beneficial effects on glucose metabolism and insulin action. Research indicates that IP6 increases glucose uptake, enhances the expression GLUT4 and IRS-1 mRNA, and promotes phosphorylation of IRS-1 [120]. These effects suggest that IP6 plays a role in enhancing insulin sensitivity in adipocytes, potentially through mechanisms involving GLUT4 translocation and IRS-1 activation. Furthermore, IP6 exhibits insulin-mimetic properties that affect metabolic pathways beyond glucose uptake. In hepatocytes, IP6 decreases the expression and transcription rate of the phosphoenolpyruvate carboxykinase (PEPCK) gene, which is crucial for gluconeogenesis [121]. Additionally, IP6 influences cellular processes involved in vesicle trafficking, impacting both exocytosis and endocytosis in eukaryotic cells [122].

Chronic inflammation induces the activation of stress kinases such as JNK and IKK, which can serine phosphorylate IRS proteins, leading to impaired insulin signaling. Endoplasmic reticulum stress results in the activation of the unfolded protein response, which can also interfere with insulin signaling through similar mechanisms [123,124,125]. Taken together, these findings suggest that enhancing Akt activation may represent a promising strategy to ameliorate insulin resistance, offering an alternative approach to current treatments. Further studies are needed to identify the molecular mediators involved in all phases of insulin-stimulated glucose uptake.

## 5. Cardiovascular Manifestations of Insulin Resistance

The main disease associated with insulin resistance and alterations of the insulin cardiac axis is diabetic cardiomyopathy, particularly pathological cardiac hypertrophy. The primary manifestations of diabetic cardiomyopathy include hypertrophy, fibrosis, cardiac diastolic dysfunction, and systolic heart failure [126]. Cardiac metabolic disturbances, autonomic dysfunction, subcellular signaling abnormalities, activation of the renin–angiotensin–aldosterone system, inflammation, maladaptive immune response, and oxidative stress are the main pathophysiological disorders linked to insulin resistance and the development of diabetic cardiomyopathy [127]. 

### 5.1. Cardiac Metabolic Disturbances

In conditions of insulin resistance, associated with an increase in carbohydrate and fat intake, the reduction in cellular ATP production triggers a state of increased ROS production, leading to intracellular oxidative damage within mitochondria [98,128,129]. The oxidative stress at the mitochondrial level induces alterations in the functionality of the sarcoplasmic reticulum, resulting in increased proteasome degradation of incorrectly folded proteins [130,131]. Alterations in mitochondrial function and the sarcoplasmic reticulum therefore induce changes in the functionality of membrane proteins that manage calcium handling. Impaired calcium handling is associated with reduced intracellular calcium uptake and delayed diastolic relaxation. Along with ROS production and endoplasmic reticulum stress, impaired calcium handling promotes subcellular component dysfunction, ultimately causing apoptosis, necrosis, and autophagy [132,133,134].

Under normal conditions, insulin and myocardial insulin signaling stimulate the translocation of GLUT4 and CD36 to the myocyte sarcolemma to supply myocardial energy substrates. However, in a state of insulin resistance, GLUT4 is internalized into cells, resulting in a reduction in energy intake from glucose and increased free fatty acid oxidation, leading to reduced cardiac efficiency [135,136,137,138]. 

### 5.2. Autonomic Dysfunction

Diabetes often leads to neurosensorial damage and neuropathy [139,140]. Specifically, diabetic Cardiac Autonomic Neuropathy (CAN), even in the absence of cardiac disease, appears linked to both left ventricular systolic and predominantly diastolic dysfunction, although isolating its independent role among the numerous factors involved in diabetic cardiomyopathy is challenging [141].

Initially marked by parasympathetic denervation, early stages of diabetic CAN may promote left ventricular hypertrophy due to excessive sympathetic activation, thereby affecting sympathovagal balance and baroreflexes [142]. Additionally, abnormal signaling of norepinephrine may induce myocardial injury and left ventricular remodeling through the cytotoxic effects of heightened catecholamine levels observed in diabetic rat hearts [143]. These effects are potentially mediated by oxidative stress, inflammation, and apoptosis [144,145,146].

Conversely, prolonged diabetic CAN-associated sympathetic denervation may impair β-adrenergic signaling, reducing myocardial contractile strength, relaxation kinetics, and diastolic distensibility [147,148,149]. Alterations in myocardial neurotransmitters due to CAN can also impair myocardial blood flow and directly worsen left ventricular function [150]. In fact, hyperactivation of the sympathetic nervous system induces vessel wall changes, promoting myocyte hypertrophy and apoptosis, interstitial fibrosis, and reduced contractile function [151]. Additionally, hyperinsulinemia can contribute to hypertension through mechanisms such as sodium retention, which increases blood volume and further exacerbates cardiovascular stress. The combined effects of sympathetic denervation and hyperinsulinemia-driven sodium retention not only exacerbate hypertension but also worsen cardiac hypertrophy [152].

Early onset of diastolic dysfunction linked to abnormal cardiac sympathetic function is observed in type 1 diabetes mellitus, as evidenced by cardiac sympathetic imaging [153]. Among individuals with type 2 diabetes mellitus or impaired glucose tolerance referred for elective coronary angiography, those with CAN exhibit a higher prevalence and more severe forms of left ventricular diastolic dysfunction [154]. In a large cohort study using cardiac magnetic resonance imaging in patients with type 1 diabetes mellitus, CAN is associated with increased left ventricular mass and concentric remodeling [155].

### 5.3. Subcellular Signalling Abnormalities

Peroxisome proliferator-activated receptor-α (PPARα) and peroxisome proliferator-activated receptor gamma coactivator 1α (PGC-1α) are important signal transduction molecules involved in regulating β-oxidation of fatty acids. This pathway modulates the mitochondrial citric acid cycle and electron transport chain. Moreover, the PGC-1β/PPARα pathway is involved in regulating cardiac metabolism, particularly in conditions of insulin resistance. Preclinical studies in mice have shown that both genetic modulation and protein concentrations of PPARα and PGC-1α are impaired in insulin resistance conditions, leading to an impairment of the insulin–cardiac axis [156,157]. Specifically, in conditions such as heart failure and diabetic cardiomyopathy, PPARα gene expression is downregulated, and the concentrations of PPARα and PGC-1α are reduced. These alterations contribute to metabolic stress, characterized by a reduction in protective substrates, ultimately leading to a greater predisposition to heart failure and diabetic cardiomyopathy [158,159,160,161,162].

### 5.4. Renin–Angiotensin–Aldosterone System

The renin–angiotensin–aldosterone system (RAAS) significantly influences insulin resistance (IR) and its complications, particularly in hyperglycemic conditions [163]. Despite salt and volume excess, RAAS activation is implicated in diabetic cardiomyopathy, while blocking RAAS has been shown to mitigate cardiac damage [164]. In addition to its AT1 and AT2 receptors, angiotensin II (Ang-II) interacts with NOX, leading to increased oxidative stress and inflammation through enhanced production of oxidants and free radicals [165]. Studies confirm the effectiveness of drugs like ramipril in reducing markers of oxidative stress such as p47phox and p22phox, and in inhibiting NADPH-driven oxidant production [166,167].

RAAS activation not only induces systemic insulin resistance but also affects cardiac insulin signaling pathways, including the mTOR–S6K1 pathway [168]. Furthermore, heightened signaling through Ang-II type 1 receptors and mineralocorticoid receptors in myocardial cells promotes a proinflammatory immune response characterized by increased leukocyte adhesion, cytokine expression, and macrophage infiltration, contributing to chronic cardiac inflammation [169]. In addition to impairing insulin signaling, activation of mineralocorticoid receptors via RAAS triggers the mTOR-S6K1 pathway, which reduces nitric oxide production in cardiovascular tissues. This reduction impairs cardiac relaxation and, over time, leads to vascular and cardiac stiffness, ultimately causing cardiac fibrosis and diastolic dysfunction [170,171,172].

### 5.5. Inflammation and Maladaptive Immune Response

In conditions of insulin resistance and diabetic disease, both the innate and adaptive immune systems play a fundamental pathophysiological role in the modulation and progression of inflammatory damage that develops at the endothelial level [173]. Moreover, cardiac insulin signaling also appears to be altered by the pro-inflammatory response [174]. Helper T cells seem to be activated, and concomitant activation of macrophage cells is associated with inflammatory cell proliferation and an increase in inflammatory cytokine secretion, leading to impaired insulin signaling [175,176,177]. The activated T cells could modulate increased cytokine secretion, and the resultant milieu of pro-inflammatory cytokines, chemokines, and growth factors is an important risk factor for cardiac fibrosis and impaired diastolic relaxation [178]. 

Additionally, PPARα’s role in modulating the inflammatory response, mediated by the inhibition of nuclear factor kappa-light-chain-enhancer of activated B cells (NF-κB) activity, seems to be impaired in this context [179]. NF-κB-regulated genes are overexpressed in these individuals, and the consequent activation of NF-κB in myocardial tissue appears to contribute to the progression of inflammation in myocardial muscle cells in subjects with heart failure [180].

## 6. Cardiovascular Diseases

**Figure 3:**
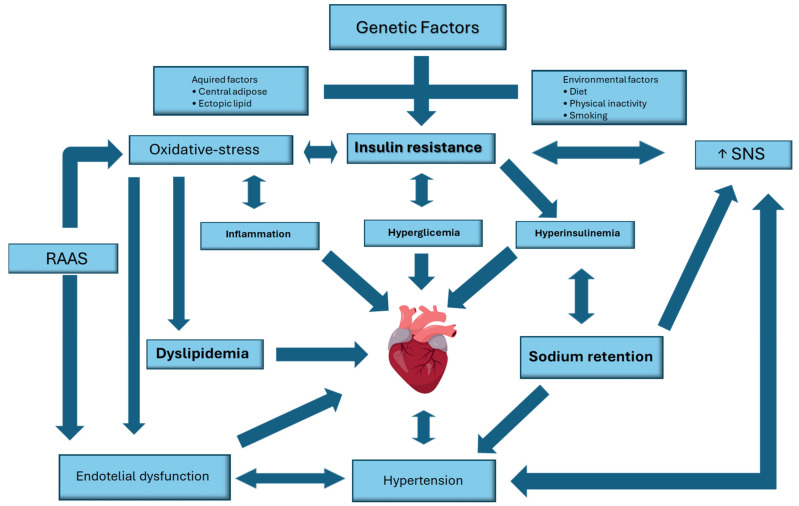
Modifications in insulin resistance pathways contributing to cardiovascular disease risk. SNS: sympathetic nervous system, RAAS: renin–angiotensin–aldosterone system.

As described above, the insulin–heart axis is pathologically associated with the development of heart diseases, primarily cardiac hypertrophy and diabetic cardiomyopathy. However, alterations in the insulin–heart axis could also be risk factors for other heart pathologies and foster additional risk factors [99] (Figure 3).

### 6.1. Epidemiology Variations in the Context of Diabetes and Cardiovascular Disease Risks

#### 6.1.1. Variations by Age

Age is a critical factor influencing the prevalence and severity of insulin resistance and associated cardiovascular disease risks. As individuals age, there is a natural decline in insulin sensitivity, which can be exacerbated by age-related increases in adiposity, changes in body composition, and reductions in physical activity. Older adults often experience a higher accumulation of visceral fat, which is strongly associated with IR [181]. Additionally, aging is linked to a decline in β-cell function in the pancreas, resulting in reduced insulin secretion. The combination of these factors contributes to a higher incidence of T2DM and cardiovascular disease in the elderly [181,182]. Studies have shown that interventions such as maintaining a healthy diet, engaging in regular physical activity, and managing body weight are crucial in mitigating the age-related decline in insulin sensitivity and reducing the risk of diabetes and cardiovascular complications [183].

#### 6.1.2. Variations by Sex

Research indicates that premenopausal women have a notably lower incidence of metabolic disorders, including IR, compared to men [77]. This is largely due to the protective effects of female sex hormones like estradiol (17β-oestradiol) that enhance the activity of proopiomelanocortin neurons and link insulin receptors to transient receptor potential channel activation, thereby guarding against IR [184,185,186]. Endogenous estrogens also activate estrogen receptor-α in various tissues, such as the brain, liver, skeletal muscle, and adipose tissue, which helps reduce IR. Additionally, estrogens impact body fat levels, fat distribution, glucose metabolism, and insulin sensitivity, leading to higher insulin responsiveness in premenopausal women than in men [181,187,188]. Differences in visceral and hepatic fat, lower levels of the insulin-sensitizing hormone adiponectin, resting energy expenditure, and lipid metabolism contribute to higher IR in males compared to females, highlighting the need for more research into these sex-specific mechanisms [189,190].

#### 6.1.3. Ethnic Variations

Insulin sensitivity and resistance exhibit significant variation across different ethnic groups. In Asia, the Singapore Adults Metabolism Study found that Chinese and Malays had higher insulin sensitivity compared to Asian Indians among lean, young Singaporean males, correlating with a lower prevalence of T2DM in Chinese (9.7%) and Malays (16.6%) compared to Asian Indians (17.2%) [77]. In the UK, South Asian children exhibit greater insulin resistance than white European children, with girls showing more insulin resistance than boys, indicating both sex and ethnic differences in insulin sensitivity and body composition [191,192]. In the USA, African Americans consistently show greater insulin resistance compared to non-Hispanic Whites, even when factors such as fat mass and visceral adipose tissue are accounted for. Studies among adolescents reveal that African Americans have higher insulin resistance as measured by intravenous glucose tolerance tests compared to Hispanics and non-Hispanic Whites, with differences persisting despite similar body composition and abdominal adiposity [193]. In prepubertal children, African Americans displayed 42% lower insulin sensitivity than non-Hispanic Whites, with obesity, visceral fat, and ethnicity being independent risk factors [194]. Furthermore, healthy African Americans adults with a family history of diabetes show significantly lower insulin sensitivity than their non-Hispanic Whites counterparts, highlighting ethnicity as a major determinant of peripheral insulin sensitivity and greater hepatic glucose output [195]. Additionally, a meta-analysis of 48 studies confirmed the finding of greater insulin resistance in healthy African Americans compared to non-Hispanic Whites. Conversely, while Hispanic Americans exhibit similar insulin resistance to African Americans, they do not experience the same degree of clinical disparities in diabetes and cardiovascular risk, suggesting that other factors contribute to the racial disparity in T2DM among African Americans [196].

### 6.2. Impact of Insulin Resistance on Cardiac Remodeling and Hypertrophy

Cardiac remodeling, characterized by myocardial fibrosis and ventricular remodeling, underlies the development of cardiac hypertrophy and precedes the onset of heart failure [197,198]. The cardiac–insulin axis plays a pathophysiological role through both intracellular and extracellular mechanisms. Cell death, induced by apoptosis and autophagy, is fundamental to the development of cardiac hypertrophy. In a state of insulin resistance, these processes are enhanced, mainly through the inhibition of the PI3K/Akt signaling pathway, while the RAS/MAPK signaling pathway remains activated [199]. Mature cardiomyocytes are non-regenerative; therefore, the increase in apoptosis and autophagy stimulates cardiac cell hypertrophy [200,201,202].

A recent study evaluated the association between cardiac hypertrophy and insulin resistance in mice subjected to abdominal aortic constriction surgery [203]. After 20 weeks of follow-up, echocardiographic examination revealed increased parietal thickness and diastolic pressure indicative of cardiac hypertrophy. Cardiac insulin resistance was assessed through a glucose clamp test and evaluation of myocardial glucose uptake after insulin infusion. Mice with cardiac hypertrophy exhibited a higher degree of insulin resistance than the control group, as demonstrated by lower glucose uptake after insulin infusion. The same study showed that p38 MAPK protein expression was lower in the experimental group, indicating reduced mitochondrial biosynthesis and oxidative dysfunction associated with PGC-1 via post-transcriptional regulation [204,205]. Clinical studies have also demonstrated the association between insulin resistance and cardiac hypertrophy. A recent case-control study on 52 asymptomatic, Black, sub-Saharan African hypertensive subjects found that obesity and insulin resistance are primary predictors of left ventricular hypertrophy [206]. Another study on a population of 1476 subjects naïve to clinical cardiovascular disease evaluated the association of insulin resistance with heart remodeling. Cardiovascular magnetic resonance imaging revealed that insulin resistance was associated with cardiac hypertrophy, reduced myocardial shortening, and torsion [207]. Cardiac remodeling associated with insulin resistance also appears to involve the right heart chambers and results in progressive alterations in filling pressures and volumes, contributing to heart failure risk [208]. A sub-analysis of the TOSCA study, conducted on patients with type 2 diabetes mellitus over 36 months, showed a higher risk of death and hospitalizations associated with echocardiographic evaluation [209]. Another clinical study on subjects with heart failure with preserved ejection fraction found a significant association between insulin resistance and myocardial dysfunction, specifically worse left ventricular longitudinal strain, even after multivariable adjustment (*p* = 0.040) [210]. Intriguingly, it was observed in vivo that insulin induces a significant rise in LVEF after submaximal work. However, this rise was significantly lower in insulin-resistant subjects, both with type 2 diabetes [211], and in obese non-diabetic subjects [212]. Furthermore, insulin resistance has been associated with coronary artery disease [213]. In Denmark, nearly 5000 subjects who underwent coronary angiography were screened for a genetic risk score of 53 single nucleotide polymorphisms for cardiovascular diseases [214]. A strong association was found between genetic predisposition to insulin resistance and coronary artery disease (OR 1.41, 95% CI: 1.10–1.82, *p* < 0.01) [214]. 

### 6.3. The Role of Insulin Resistance in Altered Lipid Metabolism and Cardiovascular Risk

The altered lipid profile caused by insulin resistance typically includes hypertriglyceridemia, increased concentrations of very-low-density lipoprotein (VLDL), decreased concentrations of high-density lipoprotein (HDL), and the formation of small dense LDL (sdLDL) [215]. Insulin regulates lipid metabolism at multiple levels. VLDL is synthesized in the liver through a process regulated by insulin, which promotes the degradation of apoprotein-B (apoB) via the PI3K pathway, hindering VLDL assembly and secretion. In insulin resistance, this mechanism is altered, resulting in increased production of triglyceride-rich VLDL [216]. Additionally, reduced lipoprotein clearance contributes to high circulating levels of these particles [217]. The formation of sdLDL is influenced by the altered function of cholesteryl ester transfer protein (CETP) and hepatic lipase. CETP promotes triglyceride transfer from VLDL to LDL and HDL, increasing their triglyceride content. These triglyceride-rich lipoproteins become substrates for hepatic lipase, which removes triglycerides, transforming LDL into sdLDL and increasing HDL catabolism by reducing apoprotein A (apoA) concentration [218,219]. This lipid profile is strongly atherogenic as sdLDL penetrates the vascular wall more easily, has a longer half-life, is more oxidizable, and has a lower affinity for LDL receptors [220]. Several studies point to a relationship between insulin resistance and hypertension [221,222] with numerous alterations synergistically damaging endothelial function and altering the balance between vasoconstrictor and vasodilator mechanisms [223,224]. Furthermore, hypertriglyceridemia promotes hyperactivation of the RAAS, which promote diabetic cardiomyopathy, as previously described [223].

### 6.4. The Impact of Insulin Resistance on Vascular Health and Abdominal Aortic Aneurysm Risk

Diabetes and insulin resistance exert multifaceted and detrimental effects on the vascular system, often leading to endothelial dysfunction, increased arterial stiffness, and accelerated atherosclerosis. Endothelial dysfunction, a hallmark of diabetes and insulin resistance, is characterized by impaired nitric oxide bioavailability, increased oxidative stress, and inflammatory responses within the vascular endothelium [129]. This dysfunction contributes to increased arterial stiffness, which is further exacerbated by hyperglycemia-induced cross-linking of collagen and other extracellular matrix components, making the arteries less compliant and more prone to injury [225]. Additionally, insulin resistance is associated with dyslipidemia, including elevated levels of triglycerides, LDL cholesterol, and decreased HDL cholesterol, as previously treated, which accelerates the formation of atherosclerotic plaques [215]. These plaques narrow the arterial lumen, restrict blood flow, and can lead to serious cardiovascular events such as myocardial infarction and stroke [226]. These conditions are generally associated with a higher risk of cardiovascular diseases [227]. However, intriguingly, diabetes appears to be associated with a lower risk of abdominal aortic aneurysm (AAA) [228]. The mechanisms underlying this paradoxical association are not fully understood, but several hypotheses have been proposed. One significant factor is the role of hyperglycemia in enhancing the cross-linking of collagen within the arterial wall through the formation of advanced glycation end-products (AGEs). This increased collagen cross-linking can lead to a stiffer and more structurally stable arterial wall, which may reduce the likelihood of aneurysm formation and expansion. Additionally, AGEs can inhibit the activity of matrix metalloproteinases (MMPs), enzymes that degrade extracellular matrix components and are implicated in the pathogenesis of AAA. By reducing MMP activity, AGEs may further contribute to the stabilization of the aortic wall in diabetic individuals. Moreover, some diabetes medications, such as metformin, have been shown to possess anti-inflammatory properties. Chronic inflammation is a key factor in the development and progression of AAAs, and the anti-inflammatory effects of these medications may help to mitigate this risk [229]. Metformin, in particular, has been noted for its ability to improve endothelial function and reduce oxidative stress, which could further protect against aneurysm formation [228]. Additionally, insulin resistance and hyperinsulinemia associated with type 2 diabetes can lead to increased levels of insulin-like growth factors, which may promote vascular smooth muscle cell proliferation and contribute to the strengthening of the arterial wall [230]. This proliferative effect could counteract the typical weakening of the aortic wall observed in AAA. Despite the general negative impact of diabetes on vascular health, these specific mechanisms appear to confer a protective effect against the development of AAA. However, it is important to note that while diabetes may reduce the risk of AAA, it does not eliminate it entirely [228].

## 7. Cardiovascular Advantages of Insulin Therapy

Exogenous insulin has emerged as a potential player in cardiovascular health, offering a range of benefits through various mechanisms, as evidenced by both experimental models and clinical applications. However, the effectiveness and safety of insulin therapy in improving cardiovascular outcomes require careful consideration of its benefits and potential risks [231].

### 7.1. Experimental Models

In experimental settings, insulin’s cardiovascular benefits are primarily linked to its vasodilatory and antiatherogenic effects. Insulin exerts potent vasodilatory effects by stimulating the production of nitric oxide (NO), a crucial vasodilator that reduces vascular resistance and enhances blood flow in both peripheral and coronary circulations [232,233,234]. This vasodilatory effect is mediated through the activation of endothelial NO synthase (eNOS), which is facilitated by the PI3K signaling pathway [235,236]. In animal models, insulin has been shown to improve blood flow by increasing capillary recruitment and enhancing vasodilation in response to physiological stimuli [235,236]. In addition to its vasodilatory properties, insulin has demonstrated antiatherogenic effects in experimental models. Studies have shown that insulin administration reduces the size of aortic atherosclerotic lesions in genetically modified mice, such as apolipoprotein E (ApoE) knockout mice, which are prone to atherosclerosis [237]. Furthermore, the conditional deletion of insulin receptors in these models results in accelerated atherosclerosis, underscoring the importance of insulin signaling in maintaining vascular health [238]. Insulin’s ability to modulate vascular inflammation is another critical factor. It reduces the expression of proinflammatory cytokines and transcription factors, such as nuclear factor-kappa B (NF-κB), which are involved in chronic inflammation and atherosclerosis. By decreasing oxidative stress and inflammatory markers, insulin helps mitigate the risk of cardiovascular diseases associated with chronic inflammation [239,240].

### 7.2. Clinical Setting

Clinical evidence further supports the cardiovascular benefits of insulin therapy, particularly in the management of diabetes. The Diabetes Control and Complications Trial (DCCT) and its long-term follow-up, the Epidemiology of Diabetes Interventions and Complications (EDIC) study, provide robust evidence that intensive insulin therapy improves glycemic control and reduces cardiovascular events in individuals with type 1 diabetes. This reduction in cardiovascular risk is attributed to better overall glycemic control, which helps prevent endothelial dysfunction and arterial damage [241,242]. However, the clinical application of insulin is not without challenges. Intensive insulin therapy is often associated with an increased risk of hypoglycemia, which has been linked to adverse cardiovascular outcomes. The ACCORD and ADVANCE trials highlighted that severe hypoglycemia could lead to a two- to threefold increase in cardiovascular events and mortality. This risk underscores the need for careful monitoring and management of blood glucose levels to prevent hypoglycemic episodes [243,244]. In addition to its role in managing blood glucose levels, insulin has antiplatelet effects that are beneficial in preventing cardiovascular events. In healthy males, insulin reduced platelet aggregation in response to various agonists by producing NO and cGMP [245,246]. Insulin reduces platelet aggregation and hyperactivity, which is particularly relevant in the context of acute coronary syndromes [247]. Finally, insulin therapy has also been shown to impact carotid intima-media thickness, a surrogate marker for atherosclerosis. Intensive insulin therapy can significantly slow the progression of this marker in patients with type 1 diabetes, indicating a potential protective effect against atherosclerosis. Additionally, insulin’s ability to modulate inflammation and oxidative stress in clinical settings adds to its cardiovascular benefits, making it a valuable component of diabetes management [248].

## 8. Future Directions

Future research should focus on elucidating the precise molecular mechanisms underlying the insulin–cardiac axis and its cardiovascular manifestations. Investigating novel therapeutic targets, such as specific signaling pathways and molecular mediators involved in insulin-stimulated glucose uptake and cardiac insulin signaling, holds promise for developing more effective treatments. In particular, research into GLP-1 receptor agonists, as well as GLP-1/GIP and GLP-1/GIP/glucagon agonists, could enhance understanding of their dual role in improving insulin sensitivity and conferring cardiovascular benefits through endothelial function improvement and inflammation reduction [249]. Similarly, SGLT2 inhibitors, which offer cardiovascular protection beyond glycemic control by lowering blood pressure and optimizing myocardial metabolism, are crucial areas of study [250]. Exploring adiponectin mimetics, such as AdipoRon, that replicate adiponectin’s anti-inflammatory and anti-atherogenic effects could also yield significant therapeutic insights [251]. Additionally, addressing the role of resistin and developing antagonists to counteract its adverse cardiovascular impacts remains a vital area for research [252]. Moreover, exploring the genetic and epigenetic factors contributing to insulin–cardiac axis and its complications could provide insights into personalized medicine approaches [253]. Longitudinal studies are needed to better understand the progression of insulin resistance-related cardiovascular diseases and the impact of early interventions, as well as the impact of a multifactorial approach and in special populations [254,255,256,257]. Lastly, clinical trials evaluating the efficacy and safety of new pharmacological agents targeting insulin–cardiac axis and its downstream effects on the cardiovascular system are crucial [258].

## 9. Research Methodology

To investigate the insulin–heart axis, we conducted a comprehensive literature search in PubMed, Scopus, and Web of Science (last accessed on 24 July 2024) using keywords such as “insulin signaling”, “heart”, “cardiomyocytes”, “metabolic dysfunction”, “cardiovascular disease”, and related terms. Our search included articles published since June 2024, focusing on peer-reviewed studies in English. From an initial pool of more than 2500 articles, we screened titles and abstracts, excluding articles not meeting our inclusion criteria (studies focusing on the physiological role of insulin in cardiomyocytes, research addressing the pathological implications of disrupted insulin signaling in cardiovascular diseases, experimental studies, clinical trials, and observational studies that provide insights into the insulin–heart axis). The full texts of about 1000 articles were assessed, leading to the selection of 258 articles relevant to the physiological and pathological roles of insulin signaling in the heart. We synthesized data from these studies to highlight key mechanisms and therapeutic implications, while noting the limitation of excluding non-English articles.

## 10. Conclusions

Insulin resistance is a multifaceted condition with significant implications for cardiovascular health. It involves complex interactions between insulin signaling pathways, metabolic disturbances, autonomic dysfunction, subcellular signaling abnormalities, RAAS activation, and inflammation. These processes contribute to the development of diabetic cardiomyopathy, cardiac hypertrophy, and other cardiovascular diseases. Understanding the underlying mechanisms and identifying effective therapeutic targets are essential for mitigating the adverse cardiovascular effects of insulin resistance.

## Data Availability

No dataset was generated for the publication of this article.
